# Vegetation–edaphic correlation and importance value index in himalayan ‘ecotone’ temperate conifer forest using the multivariate technique

**DOI:** 10.1016/j.sjbs.2024.103983

**Published:** 2024-03-24

**Authors:** F. Ali, M. Zeb, M. Amin, M.N. Rajpar, S. Hidayat, W.R. Khan

**Affiliations:** a Department of Botany, Faculty of Life Sciences, Shaheed Benazir Bhutto University Sheringal, Dir Upper 18050, Pakistan; b Department of Forestry, Faculty of Life Sciences, Shaheed Benazir Bhutto University Sheringal, Dir Upper 18050, Pakistan; c Department of Environment, Faculty of Life Sciences, Shaheed Benazir Bhutto University Sheringal, Dir Upper 18050, Pakistan; d Department of Forestry Science, Faculty of Agricultural and Forestry Sciences, Universiti Putra Malaysia Kampus Bintulu Sarawak, 97008, Malaysia; e Institut Ekosains Borneo, Universiti Putra Malaysia Kampus Bintulu Sarawak 97008, Malaysia

**Keywords:** Ecotone, Importance value index, Himalayan conifer, Floral structure, Multivariate CCA

## Abstract

Himalayan ‘Ecotone’ temperate conifer forest is the cradle of life for human survival and wildlife existence. In spite of the importance of these areas, they have not been studied in depth. This study aimed to quantify the floristic structure, important value index (IVI), topographic and edaphic variables between 2019 and 2020 utilizing circular quadrant method (10 m x 10 m). The upper-storey layer consisted of 17 tree species belongs to 12 families and 9 orders. Middle-storey shrubs comprised of 23 species representing 14 families and 12 orders. A total of 43 species of herbs, grasses, and ferns were identified from the ground-storey layer, representing 25 families and 21 orders. Upper-storey vegetation structure was dominated by *Pinus roxburghii* (22.45 %) and middle-storey by *Dodonaea viscosa* (7.69 %). However, the ground layer vegetation was diverse in species composition (43 species) and distribution. The floral vegetation structure was encompassing of three floral communities which were diverse in IVI, such as, in Piro–Aial (Group 2), *Pinus roxburghii* (54.46 x 15.94) had the highest IVI value, followed by *Pinus wallichiana* (45.21 x 14.85) in Piwa–Quin (Group 3) and *Ailanthus altissima* (22.84 x 19.25) in Aial–Qugal (Group 1). However, the IVI values for *Aesculus indica*, *Celtis australis*, and *Quercus incana* in Aial–Qugal (Group 1) were not determined due to low detection rate. Nevertheless, eleven of these species showed 0 IVI values in Piro–Aial (Group 2) and Piwa–Quin (Group 3). CCA ordination biplot illustrated the significant differences among floral communities and its distribution, which impacted by temperature, rainfall, soil pH, altitude, and topographic features. Ward's agglomerative clustering finding reflected 'Ecotone' temperate conifer forest is rich and diverse floristic structure.

## 1 Introduction

Geographically and topographically, Himalayan ‘Ecotone’ temperate conifer forest is diverse in landscape features as well as floral structure and composition. Forests are vital for life on the planet and cover 31.0 % of the world's land surface, acting as an important buffer against climate change (Moomaw et al., 2020) and providing a wide range of services for the well–being of humans (Sohail et al., 2023) and wildlife (Zhang and Li, 2016). Forests have diverse vegetation structure and composition, providing ample benefits, i.e., food, shelter, breeding and foraging grounds to wildlife species around the world. Climatic, edaphic and topographic features play a crucial role in forest development and distribution (Ali and Khan, 2022, Ali et al., 2022). A variety of forest types are constituted due to these factors, which allow diverse fauna species to thrive and maintain their existence (Gong et al., 2021).

Himalayan ‘Ecotone’ temperate conifer forest is the transition area between moist-temperate and dry-temperate conifer forest. This region has complex, diverse, and rich vegetation. The structure and composition of vegetation reflect productivity, habitat suitability, ecological balance, and the integrity of ecological ecosystem, i.e., it provides ample services for human well–beings (Hou et al., 2019). Environmental factors (Yasmeen et al., 2023) and ecosystem services can be derived from plant species distributions. Plants play an important role in maintaining the equilibrium of the ecosystem and provide a multitude of benefits to humans and other organisms (Ali et al., 2022, Ali and Khan, 2022).

The Himalayan ‘Ecotone’ temperate conifer forests are vital for human survival as well as wildlife species. Despite their importance for survival and existence, human intervention, i.e., land use changes, i.e., conversion into agriculture and human settlements (Daye and Healey, 2015, Moisa et al., 2022), deforestation (Lawrence and Vandecar, 2015, Liao et al., 2018), and climate change (Akram et al., 2022, Gemeda et al., 2022, Khan et al., 2021) have contributed to a decrease in two-thirds of forest covers over the past two decades (Miyamoto et al., 2014, Alkama and Cescatti, 2016, Tsegaye et al., 2023). It is therefore imperative to quantify the relationship between floristic composition, edaphic variables, and environmental determinants in order to conserve, protect, and sustainably manage forests for the present and for the future. It is important to interpret plant species from any geographical location in terms of floristic characteristics, IVI value, and correlation between vegetation, edaphic, and climatic conditions (Hanz et al., 2022, Manan et al., 2022). As a result, floristic composition may be influenced by various factors, including climate, soil type, topography, human activities, and biotic interactions (Aslam et al., 2022, Abedi et al., 2022, Bisht et al., 2022).

Variations in vegetation are largely influenced by climate factors like temperature, precipitation, and human interference (Anees et al., 2022a, Ali et al., 2017). Climate change is causing vegetation to respond in a response that is extremely important to emphasize (Usoltsev et al., 2020, Andreevich et al., 2020, Shobairi et al., 2022, Usoltsev et al., 2022). Among the most important environmental factors affecting vegetation are temperature and precipitation (Ali et al., 2023a, Anees et al., 2022b). In particular, these factors affect the distribution of plant species, defining the boundaries between deserts, grasslands, and forests (Burruss et al., 2023). These factors also affect plant growth and development, and different species tolerate them differently (Pan et al., 2023).

Geographical differences in vegetation are largely influenced by climate factors like temperature, precipitation (Anees et al., 2024), and even human interferences (Ali et al., 2017). It is very important to highlight the response of vegetation to the changing climate (Mehmood et al., 2024b). Temperature and precipitation are among the most prominent environmental determinants for vegetation (Ali et al., 2023b, Mehmood et al., 2024a). These factors play a crucial role in determining the distribution of plant species, particularly in determining the boundaries of biomes such as deserts, grasslands, and forests (Burruss et al., 2023). It also affects growth and development and plant species exhibits varying tolerances to these factors. For example, some plant species may be adapted to thrive in arid environments, while others may require higher levels of precipitation to grow and reproduce. Some plant species are capable of growing in arid environments, while others need higher levels of precipitation to thrive.

In transitional zones of any forest ecosystem, no detailed study has been conducted to determine the correlation and importance value index. In different regions around the world, only a few studies have explored how environmental variables influence vegetation cover and floristic composition (Khan et al., 2015, Ali et al., 2017, Ali et al., 2022, Ali and Khan, 2022). Detailed information about how climate, environment, and topography affect floristic characteristics in Himalayan ‘Ecotone’ temperate conifer forest is lacking in Pakistan. Through the use of multivariate techniques, the main objective of this study was to quantify vegetation–edaphic correlation and importance value index to understand how multivariate variables affect floral structure and composition growth and distribution.

## 2 Materials and methods

### 2.1 Study area

**Fig. 1:**
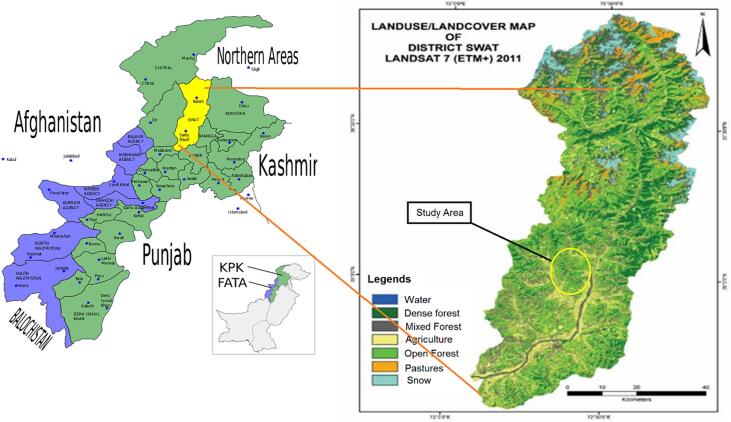
Location map of the study area (Himalayan ‘Ecotone’ temperate conifer forest).

An ecological study of vegetation structure and composition was conducted in Himalayan ‘Ecotone’ temperate conifer forest, covering a surface area of 5337 km^2^, located between 34° 0′ 34′′ and 35° 0′ 55′′ north latitude and 72° 0′ 08′′ and 72° 0′ 50′′ east longitude, Swat Pakistan (Fig. 1). This forest is a transitional area between moist-temperate and dry-temperate conifer forest. Due to its diverse vegetation structure, topographic landscapes, edaphic and climatic conditions, it has been recognized as a biodiversity hub in the Hindukhush mountain ranges (Chen et al., 2023). The altitude ranged from 3500ft to 10,000 masl. Higher elevations are dominated by *Pinus wallichiana, Quercus delatata, Q.* semicarpifolia, *Fraxinus hookeri*, *Aesculus indica*, and shrub species, i.e., *Berberis lyceum*, *Viburnum cotinifolium*. In contrast, at lower elevations, tree species include *Pinus roxburgii*, *Q. incana*, *Pyrus pashia*, *Pistacia integerrima*, *Punica granatum*, and shrub species, e.g., *Rhododendron arboreum* (Rahman et al., 2021).

#### 2.1.1 Sampling design and data collection

Forest resources across the country are being severely depleted by human intervention and climate change (Ullah et al., 2023). To understand the current status, productivity and ecological importance, in total, 300 circular quadrat plots were established, which encompassing of 10 x 10 m^2^ for upper–storey (tree species), 5 x 5 m^2^ for middle–storey (shrub species), and 1 x 1 m^2^ ground–storey vegetation encompassing of herbs, grasses, and ferns were established randomly selected across the hilly terrain, foothills, riparian areas, pastures and valleys in the study areas. In addition, phytosociological variables, such as; tree biomass, height, DBH (Diameter at breast height in meters), aspect, slope, and physiochemical properties of soil were also examined to understand which variable play significant role on plant composition, structure and distribution in the study area (Qian et al., 2003, Wulf and Naaf, 2009). The methodology was followed as described by Ali et al. (2017).

Using a diameter tape, we measured the diameter at breast height of all recorded tree species within each plot. The height (m) of tree species was also measured with a telescopic Hastings fiberglass rod (H < 15 m) and Abneys level (Hou et al., 2019). Several plant species were observed to exhibit the phenomenon of multi-stem trees. In order to calculate multi-stem tree diameter, all stem diameters were measured and then divided by the number of stems (Nakhoul et al., 2020). Moreover, each target sampled plot was recorded in terms of its slope angle, elevation, and aspect. Additionally, vegetation, soil samples, and microclimate data were recorded when edaphic variables were applied. Specifically, this methodology was followed as explained by (Liu et al., 2018).

### 2.2 Data analysis

The relative abundance of the Himalayan ‘Ecotone’ temperate conifer forest was quantified by equation: Relative Species Abundance (%) = Isi/ ∑ Nsi × 100; Where, Isi = Total number of an individual plant species, / ∑Nsi = Total number of detected plant species, e.g., tree species (upper-storey layer), shrub species (middle-storey layer), and grass species (ground-storey layer).

By using the standard methodology of (Pala et al., 2020), the Importance Value index (IVI) was determined for each plant species. Multivariate analysis software, PC-ORD (6.0), was used to analyze IVI value of all species of plants using Ward's agglomerative clustering. Using Rahman et al. (2021) as a guide, we evaluated floristic composition consisting of 17 species over 150 plots and environmental variables (150 plots over 17 variables). Conical Correspondence Analysis (CCA–ordination) was used to quantify the correlation between flora and environmental variables using the multivariable (Ali et al., 2019).

The floristic structure of the study area was understood through the calculation of absolute densities ha^–^^1^ and cover m^2^ha^–^^1^. Additionally, soil parameters (pH, texture, inorganic nutrients, saturation, etc.) were evaluated in the soil chemistry laboratory of the Agriculture Research Institute (ARI), Mingora Swat (Liu et al., 2021).

## 3 Results

### 3.1 Floristic structure and layer composition

On the basis of 83 plant species detected from the study area, three stratums of vegetation were identified, i.e., upper–storey (trees), middle–storey (shrubs), and ground–storey stratum (herbs, grasses and ferns).

### 3.2 Upper–Storey layer

The upper–storey layer of 'Ecotone' temperate conifer forest floral structure was composed of 17 tree species representing 12 families and 9 orders. Long–leaf Indian pine – *Pinus roxburghii* (22.45 %) was the most dominant tree species. Contrarily, four tree species were least abundant, i.e., Himalayan horse chestnut – *Aesculus indica*, Winged prickly ash – *Zanthoxylum armatum*, Armenian plum – *Prunus armeniaca*, and Chocolate persimmon – *Diospyrus nigra* (each constituted; 0.17 % Table 1).

**Table 1:** Tree species composition (Upper–storey layer) of Himalayan ‘Ecotone’ temperate conifer forest.

Order	Family	Scientific Name	Common Name	Percentage
Coniferalus	Pinaceae	*Pinus roxburghii*	Long Leaf Indian Pine	22.45
Rosales	Fabaceae	*Robinia pseudoacacia*	Black locust	14.09
Sapindales	Simaroubaceae	*Ailanthus altissima*	Tree of Heaven	13.43
Fagales	Fagaceae	*Quercus incana*	Bluejay Oak	12.55
Rosales	Urticaceae	*Debregeasia salicifolia*	Himalayan Wild Rhea	9.28
Rosales	Moraceae	*Ficus palmata*	Punjab Fig	8.64
Pinales	Pinaceae	*Pinus wallichiana*	Himalayan White Pine	8.29
Fagales	Fagaceae	*Quercus glauca*	Ringed Cup Oak	5.14
Unisexuales	Moraceae	*Ficus glomerata*	Indian Fig Tree	3.81
Sapindales	Meliaceae	*Melia azedarach*	China Berry	0.50
Rosales	Rosaceae	*Pyrus pashia*	Wild Himalayan Pear	0.49
Rosales	Cannabaceae	*Celtis australis*	Honeyberry	0.33
Fagales	Juglandaceae	*Juglans regia*	English Walnut	0.32
Sapnidales	Sapindaceae	*Aesculus indica*	Himalayan Horse Chestnut	0.17
Geraniales	Rutaceae	*Zanthoxylum armatum*	Winged Prickly Ash	0.17
Rosales	Rosaceae	*Prunus armeniaca*	Armenian Plum	0.17
Ericales	Ebenaceae	*Diospyrus nigra*	Chocolate Persimmon	0.17

#### 3.2.1 Middle-Storey layer

The middle-storey layer comprises 23 shrub species representing 14 families and 12 orders. Hope Bush – *Dodonaea viscosa* (7.69 %) was the most abundant shrub species in the study area. Contrarily, vein leaf viburnum – *Viburnum nervosum* and coastal rosemary – *Westringia glabra* were rarest shrub species of the middle-storey layer of the research area (Table 2).

**Table 2:** Shrub species composition (Middle-storey layer) of Himalayan ‘Ecotone’ temperate conifer forest.

Order	Family	Scientific Name	Common Name	Percentage
Sapindales	Sapindaceae	*Dodonaea viscosa*	Hope Bush	7.69
Lamiales	Lamiaceae	*Teucrium fruticans*	Bush Germander	6.70
Lamiales	Lamiaceae	*Isodon rugosus*	Winkled Leaf Isodon	6.70
Polygonales	Polygonaceae	*Rumex hastatus*	Arrow leaf Dock	6.20
Rosales	Rosaceae	*Cotoneaster gracilis*	Bearberry	5.71
Rosales	Rhamnaceae	*Ziziphus nummularia*	Wild Jujube	5.71
Lamiales	Lamiaceae	*Vitex negundo*	Horseshoe Vitex	5.46
Rosales	Rosaceae	*Cotoneaster apiculatus*	Cranberry	5.21
Buxahus	Buxaceae	*Sarcococca elaeagnus*	Sweet Box	4.71
Solinales	Solanaceae	*Withania coagulans*	Indian Rennet	4.71
Solinales	Solanaceae	*Datura inoxia*	Downy Thorn Apple	4.47
Fabales	Fabaceae	*Indigofera gerardiana*	Himalayan Indigo	4.47
Ranunculales	Berberdaceae	*Berberis lycium*	Indian Barberry	4.22
Solinales	Thymelaceae	*Daphne mucronata*	Khewesk	4.22
Solinales	Solanaceae	*Withania somnifera*	Winter Cherry	4.22
Rosales	Urticaceae	*Debregeasia salicifolia*	Himalayan Wild Rhea	3.72
Asparagales	Liliaceae	*Asparagus adscendens*	West-Himalayan Asparagus	3.47
Alismatales	Asparagaceae	*Arisaema* spp.	Cobra lily	2.98
Renales	Rosaceae	*Rubus niveus*	Ceylon Raspberry	2.73
Renales	Rosaceae	*Rubus niloticus*	Blackberry	2.23
Renales	Rosaceae	*Rubus ellipticus*	Yellow Himalayan Raspberry	1.99
Bipsacles	Adoxaceae	*Viburnum nervosum*	Vein leaf Viburnum	1.24
Lamiales	lamiaceae	*Westringia glabra*	Coastal Rosemary	1.24

### 3.3 Grasses, herbs, and fern species (Ground-storey Layer)

The ground–storey layer of ‘Ecotone’ temperate conifer forest was encompassing of 43 herbs, grasses and fern species representing 25 families and 21 orders. The result shows that Bracketed Bugleweed – *Ajuga bracteosa* accounted for 4.21 % and Common Chickweed – *Stellaria media* accounted for 1.02 % of the weeds in the study area. Similarly, hemp – *Cannabis sativa* (3.70 %) was the most prevalent grass species while on the other hand, the redstem wormwood – *Artemisia scoparia* (0.51 %) was the scare grass species. Moreover, Chinese ladder brake fern – *Pteris vittata* (1.79 %) was the most common species and sword fern *Asplenium dalhousiae* (1.40 %) was the rarest species in research areas (Table 3a, Table 3b).

**Table 3a:** Grasses, herbs and fern species composition (ground-storey layer) of ‘Ecotone’ conifer temperate forest.

Order	Family	Scientific Name	Common Name	Percentage
Lamiales	Lamiaceae	*Ajuga bracteosa*	Bracketed Bugleweed	4.21
Unisexuales	Moraceae	*Cannabis sativa*	Hemp	3.70
Fabales	Fabaceae	*Trifolium resupinatum*	Person clover	3.58
Oxalidales	Oxalidaceae	*Oxalis corniculata*	Creeping wood sorrel	3.45
Asterales	Asteraceae	*Taraxacum officinale*	Common Dandelion	3.45
Rosales	Fabaceae	*Trifolium repens*	Clover	3.32
Caryophyales	Amaryllidaceae	*Chenopodium murale*	Nettle-leaved Goose Foot	3.19
Asterales	Asteraceae	*Sonchus asper*	Spiny Sowthistle	3.19
Asterales	Asteraceae	*Xanthium strumarium*	Rough Cocklerbur	3.19
Asterales	Asteraceae	*Conyza aegyptiaca*	Horse weed	3.07
Boragenalis	Boragenaceae	*Cynoglossum officinale*	Houndtooth	3.07
Asterales	Asteraceae	*Sonchus oleraceus*	Common Sowthistle	2.94
Ericales	Balcemenaceae	*Impatiens minima*	Jewelweed	2.81
Caryophyales	Amaryllidaceae	*Achyranthes aspera*	Prickly Chaff Flower	2.68
Apiales	Apiaceae	*Apium vulgaris*	Ajmoda or Celery	2.68
Caryophyales	Amaryllidaceae	*Chenopodium ambrosioides*	Mexican tea	2.68
Renales	Ranunculaceae	*Ranunculus repens*	Creeping Buttercup	2.68
Fabales	Fabaceae	*Trigonella foenum-graecum*	Goat’s horn	2.68
Lamiales	Acantheaceae	*Dicliptera roxburghiana*	Magenta plant	2.55
Aygophyales	Zygophyllacee	*Tribulus terrestris*	Puncture vine	2.43
Malpighiales	Violaceae	*Viola pilosa*	Smooth-Leaf White Violet	2.43
Polypediales	Pteredaceae	*Adiantum venustum*	Himalayan Maidenhair Fern	2.17
Alismatales	Araceae	*Arisaema flavum*	Yellow Cobra Lily	2.17
Malvales	Malvaceae	*Malva parviflora*	Cheeseweed mallow	2.17
Asparagales	Amaryllidaceae	*Narcissus tazetta*	Joss Flower or Daffodils	2.17
Polypediales	Pteredaceae	*Pteris vittata*	Chinese Ladder Brake Fern	2.17
Lamiales	Scrophulariaceae	*Verbascum thapsus*	Common Mullein	2.17
Caryophyllales	Polygonaceae	*Polygonum congnatum*	Indian Knotgrass	2.04
Lamiales	Lamiaceae	*Mentha longifolia*	Asian Mint	1.92
Polypediales	Pteredaceae	*Adiantum capillus-veneris*	Southern Maidenhair Fern	1.79
Fabales	Fabaceae	*Desmodium indicus*	Threeflower Ticktrefoil	1.79
Solinales	Convolvulaceae	*Ipomoea indica*	Blue Morning glory	1.79
Caryophyllales	Polygonaceae	*Polygonum amplexicaulis*	Knotweed	1.79
Asterales	Asteraceae	*Conyza leiotheca*	Hairly Fleabane	1.66
Polypediales	Aspleniaceae	*Asplenium dalhousiae*	Sword Fern	1.40
Urticales	Utricaceae	*Urtica dioica*	Stinging Neetle	1.40
Lamiales	Lamiaceae	*Calamintha vulgaris*	Wild Basil	1.28
Rosales	Rosaceae	*Fragaria indica*	Wild Straberry	1.28
Asterales	Asteraceae	*Calendula arvensis*	Field Marigold	1.15
Geraniales	Geraniaceae	*Geranium wallichianum*	Wallich Cranebill	1.15
Lamiales	Plantaginaceae	*Plantago lanceolata*	Narrowleaf Plantain	1.02
Caryophyllales	Caryophyllaceae	*Stellaria media*	Common Chickweed	1.02
Asterales	Asteraceae	*Artemisia scoparia*	Redstem Wormwood	0.51

**Table 3b:** Using 50 vegetation sampling circular plots and 17 driving factors, Eigen-values were extracted from CCA axes are as under;

	Axis 1	Axis 2	Axis 3	P-value
Eigenvalue	0.77	0.40	0.36	0.0901
Species-environmental correlations	22.3	11.7	10.5	
Cumulative percentage explained variance of species data	22.3	34.0	44.5	
Pearson Correlation, Spp–Envt*	0.95	0.93	0.86	0.1331
Kendall (Rank) Corr., Spp–Envt	0.74	0.46	0.56	

* The correlation between the sample scores for an axis derived from species data and the sample cores derived from linear combinations of environmental variables. The value should be set to 0.000 if the axis is not canonical.

### 3.4 Vegetation community types

**Fig. 2:**
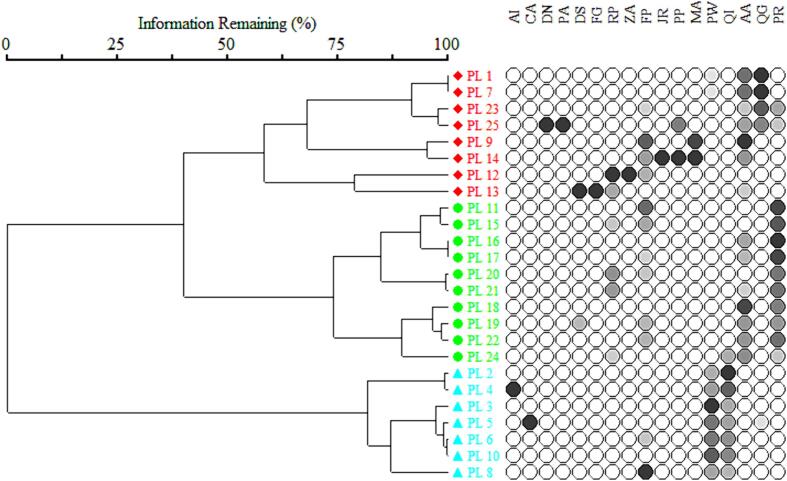
Tree dendrogram by Ward’s cluster analysis from 50 sampling sites of ‘Ecotone’ temperate conifer forest. Colors represent three different floral Community types of the study area.

Ward's agglomerative clustering analysis was used to classify tree species recorded from ‘Ecotone’ temperate conifer forest into three distinct communities, namely Community type–I, Community type–II, and Community type–III. There were 14 tree species in community type–I as determined by 8 sampling sites. The two dominant species in this community were *Ailanthus altissima* (IVI = 22.84 %) and *Quercus glauca* (IVI = 15.27 %), while other species included *Debregeasia salicifolia, Rubinia pseudoacacia, Ficus palmata*, and *Pinus roxburghii*. It was evident that these species were strongly associated with the dominant tree species. In the community type–II, six species of trees were recorded from 10 sampling sites. Among the species present in this community, *P. roxburghii* (IVI = 54.46 %) and *A. altissima* (IVI = 20.15 %) were co-dominant. In addition, prominent members of this community were *R. pseudoacacia* and *F. palmata*. Instead, *D. salicifolia* and *Q. incana* were scattered rather than distributed with dominant trees. Furthermore, IVI results indicated that the community type-III was comprised of 6 species detected at 7 sampling sites. In Community type-III, *P. wallichiana* and *Q. incana* were the most prevalent trees. The *Aesculus indica* and the *Celtis australis* were rare in this community, however. A few shrub species dominate the middle-storey layer of the floral storey, including *Berberis lyceum, Calamintha vulgaris, Cymbopogan jwarancusa*, and *Indigofera geradiana*. Consequently, based on the results of IVI, Community type–III had a lower score than Community type–I and Community type–II (Fig. 2 and Table 4).

**Table 4a:** Important Value Index (IVI) of tree species detected from ‘Ecotone’ temperate of conifer forest based on three agglomerated groups according to Ward’s agglomerative cluster analysis.

Family	Scientific Name	Common Name	Groups		
			Aial-Qugl Group 1	Piro-Aial Group 2	Piwa-Quin Group 3
Sapindaceae	*Aesculus indica*	Himalayan Horse Chestnut	0 ± 0	0 ± 0	1.36 ± 3.6
Simaroubaceae	*Ailanthus altissima*	Tree of Heaven	22.84 ± 19.25	20.15 ± 16.98	0 ± 0
Cannabaceae	*Celtis australis*	Honeyberry	0 ± 0	0 ± 0	1.43 ± 3.79
Urticaceae	*Debregeasia salicifolia*	Himalayan Wild Rhea	12.81 ± 23.97	1.6 ± 5.06	0 ± 0
Ebenaceae	*Diospyrus nigra*	Chocolate Persimmon	0.91 ± 2.59	0 ± 0	0 ± 0
Moraceae	*Ficus glomerata*	Indian Fig Tree	6.82 ± 14.96	0 ± 0	0 ± 0
Moraceae	*Ficus palmata*	Punjab Fig	9.96 ± 12.54	10.28 ± 10.48	7.64 ± 15.52
Juglandaceae	*Juglans regia*	English Walnut	3.55 ± 10.06	0 ± 0	0 ± 0
Meliaceae	*Melia azedarach*	China Berry	2.78 ± 5.16	0 ± 0	0 ± 0
Pinaceae	*Pinus roxburghii*	Long Leaf Indian Pine	6.90 ± 13.14	54.46 ± 15.94	0 ± 0
Pinaceae	*Pinus wallichiana*	Himalayan White Pine	1.21 ± 3.43	0 ± 0	45.21 ± 14.85
Rosaceae	*Prunus armeniaca*	Armenian Plum	0.85 ± 2.42	0 ± 0	0 ± 0
Rosaceae	*Pyrus pashia*	Wild Himalayan Pear	2.07 ± 3.95	0 ± 0	0 ± 0
Fagaceae	*Quercus glauca*	Ringed Cup Oak	15.27 ± 21.70	0 ± 0	1.16 ± 3.07
Fagaceae	*Quercus incana*	Bluejay Oak	0 ± 0	2.96 ± 9.38	43.18 ± 15.82
Fabaceae	*Robinia pseudoacacia*	Black locust	11.69 ± 23.79	10.52 ± 14.76	0 ± 0
Rutaceae	*Zanthoxylum armatum*	Winged Prickly Ash	2.26 ± 6.41	0 ± 0	0 ± 0

**Table 4b:** Canonical coefficients between the site–scores and twelve environmental variables obtained from CCA.

		Correlations*			Biplot Scores		
	Variable	Axis 1	Axis 2	Axis 3	Axis 1	Axis 2	Axis 3
1	Latitude	**0.668**	0.196	0.417	0.284	0.096	0.2
2	Longitude	−0.128	0.148	0.311	−0.054	0.072	0.149
3	Elevation	**−0.835**	−0.169	−0.139	−0.354	−0.083	−0.067
4	Soil temperature	**0.733**	0.129	0.228	0.311	0.063	0.109
5	Soil compaction	**0.634**	−0.033	0.072	0.269	−0.016	0.035
6	Soil moisture	**−0.564**	0.07	0.194	−0.239	0.034	0.093
7	Electrical conductivity	−0.373	−0.112	0.064	−0.158	−0.055	0.031
8	pH	−0.022	−0.067	−0.483	−0.009	−0.033	−0.232
9	Soil organic matter	−0.15	0.23	0.339	−0.064	0.113	0.163
10	Nitrogen (%)	−0.114	0.243	0.275	−0.048	0.119	0.132
11	Carbon (%)	−0.142	0.224	0.326	−0.06	0.11	0.156
12	CaCO3	−0.03	−0.116	0.214	−0.013	−0.057	0.103
13	Saturation	−0.371	0.116	−0.428	−0.157	0.057	−0.205
14	Zinc	0.066	0.074	0.201	0.028	0.036	0.097
15	Cu	0.014	0.061	−0.273	0.006	0.03	−0.131
16	Iron	0.069	0.327	**0.434**	0.029	0.16	0.208
17	Manganese	0.225	0.307	**0.41**	0.096	0.15	0.197

* Correlations are “intra–set correlations”

### 3.5 Important value index (IVI)

Floral communities showed diverse results from IVI, such as, in Piro–Aial (Group 2), *Pinus roxburghii* (54.46 x 15.94) had the highest IVI value, followed by *Pinus wallichiana* (45.21 x 14.85) in Piwa–Quin (Group 3) and *Ailanthus altissima* (22.84 x 19.25) in Aial–Qugal (Group 1). Nevertheless, none of the IVI values were determined for *Aesculus indica, Celtis australis*, and *Quercus incana* in Aial–Qugal (Group 2). In addition to Piro–Aial (Group 2) and Piwa–Quin (Group 3), zero IVI values were detected for 11 other tree species (Table 4).

#### 3.5.1 Relationship of the communities with the environmental factors

**Fig. 3:**
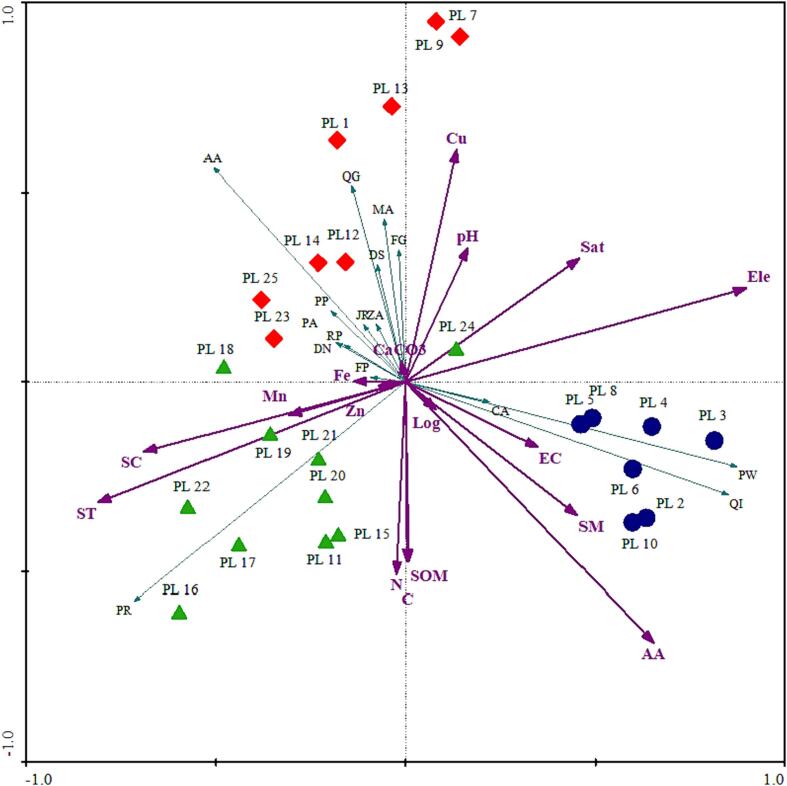
CCA biplot of 50 established sampling sites and 17 variables. Colors represent the results of three different communities.

An analysis of Canonical Correspondence Analysis (CCA) was performed to determine the correlation between trees, topographic features, and soil physicochemical properties in the 'Ecotone' temperate forest. In accordance with species-environmental correlations, the first axis can explain 22.3 % of the variable and the second axis can explain 11.7 %. Similarly, axis 1 indicates that 22.3 % of the variance of cumulative percentage can be explained and axis 2 indicates 34.0 %. Using the unrestricted Monte Carlo test permutation, the F ratios highlighted a strong correlation between the matrices, indicating that the observed patterns were not simply random (Table 3a, Table 3b and Fig. 3).

According to the CCA–ordination plot, there is a complex pattern of species composition across different environments, including latitude (r = 0.668) and elevation (r = – 0.835), which are significant on axis 1. The edaphic variables, soil temperature and soil compaction are also closely correlated with axis 1. There was a positive correlation between iron (Fe) and manganese (Mn) in axis 3 of the CCA. *P. roxburghii* occupies the negative end of axis 1 based on species biplot data. On the other hand, three species ranked at the positive end of the spectrum, namely *Q. incana, P. wallichiana*, and *C. australis.* It was observed that *A. altissima*, *F. glomerata*, *M. azedarach*, and *Q. glauca* occupies the upper portion of the CCA–biplot. A gradient of expansion along positive axes is evident here, while a gradient of shrinkage is evident along negative axes. In addition to *R. pseudoacacia, D. nigra, F. palmata*, and *M. azedarach*, and a number of other plants thrive here as well. CCA–biplot results showed that these species were grouped in the upper middle of the continuum (Fig. 3 and Table 4).

#### 3.5.2 Habitat characteristic of the communities

The sampling sites of community type–I were located at medium altitude range from 1340 m to 1849 m ($\overset{¯}{x}$ = 1513.75 masl). The physicochemical soil properties of this floral community type-1 comprised electrical conductivity (σ = 0.38 ± 0.04), soil organic matter (SOM = 0.89 ± 0.12), total nitrogen (TN = 0.04 ± 0.006), and total carbon (TC = 0.52 ± 0.07). However, the amount of iron, zinc and copper contents in the soil were founded to be higher as compared to the soil of other vegetation types (Table 5). Floral community type–II were located at the lower elevation ($\overset{¯}{x}$ = 1263.8 ± 179.98 masl), have higher soil temperature (°C = 32 ± 3.91). These attributes were more possibly due to heavy anthropogenic interferences. Similarly, sampling site of community type–III were located at steep slopes and having high mean altitudinal range ($\overset{¯}{x}$ = 1823.85 ± 54.14 masl). As the steep slope increased, the gravitational pull on soil water occurs and therefore, the soils of these sampling sites were low in moisture contents. The soil of this community was more fragile and loosely arranged indicating very less compaction (Table 5).

**Table 5:** Categorization of floral community types using Ward's agglomerative clustering method.

**Topographic Variables**		**Group 1**	**Group 2**	**Group 3**
	Latitude	34.66 ± 0.029	34.69 ± 0.027	34.64 ± 0.002
	Longitude	72.36 ± 0.013	72.36 ± 0.018	72.36 ± 0.002
	Elevation (masl)	1513.75 ± 191.65	1263.8 ± 179.98	1823.85 ± 54.14
**Physicochemical Edaphic Variables**		**Group 1**	**Group 2**	**Group 3**
	Soil Temp. (^o^C)	28.75 ± 3.45	32 ± 3.91	24.57 ± 2.43
	Soil Compaction	40.62 ± 20.6	60 ± 20.68	22.9 ± 4.34
	Soil Moisture	7.51 ± 2.13	5.32 ± 1.87	3.98 ± 1.65
	E.C. (dS/m)	0.38 ± 0.04	0.37 ± 0.04	0.49 ± 0.06
	pH	7.44 ± 0.15	7.31 ± 0.14	7.37 ± 0.19
	Soil Organic Matter	0.89 ± 0.12	1.05 ± 0.14	1.03 ± 0.2
	Total Nitrogen (%)	0.04 ± 0.006	0.05 ± 0.007	0.05 ± 0.01
	Total Organic Carbon (%)	0.52 ± 0.07	0.61 ± 0.08	0.6 ± 0.11
	CaCO_3_	5.45 ± 1.44	4.98 ± 1.45	5.35 ± 1.72
	Saturation	52 ± 3.38	49.9 ± 2.11	52.92 ± 3.48
	Zn	1.13 ± 0.31	0.9 ± 0.34	1.02 ± 0.19
	Cu	2.64 ± 0.64	1.53 ± 0.57	1.79 ± 0.92
	Fe	4.07 ± 1.32	3.56 ± 0.88	3.68 ± 0.64
	Mn	1 ± 0.38	1.03 ± 0.27	0.88 ± 0.34

### 3.6 Tree density and stand structures

In forest ecosystems, vegetation composition, species persistence, and plant richness play a significant role in determining the floral structure and layers (Augusto et al., 2003, Ali et al., 2017). The density/ha^–^^1^ and crown/ha^–^^1^ of the prevalent and associated tree species were measured. In Himalayan ‘Ecotone’ temperate conifer forest, community type–I showed the highest density of trees (245 plants/ha^–^^1^) while community type–III displayed the lowest density (26 plants/ha^–^^1^). It is noteworthy that in community type–I, *Debregeasia salicifolia* accounted for 23.98 % of the total tree density, demonstrating that this was the community's most abundant tree species. *Rubinia pseudoacacia* and *Ailanthus altissima* each contributed 15.81 % to the community's density. *Pinus roxburghii* was the most prevalent species in the type–II community, accounting for more than 45.00 % of the total density and demonstrating the most abundant tree species. *Quercus incana* (53.13 %) and *P. wallichiana* (38.39 %) were the predominant tree species in community type–III, contributing the highest percentages and ranking the highest. Furthermore, the community type–III has the highest crown cover (496.5 m^2^/ha^–^^1^), which represents 74.00 % of the community's vegetation structure. Moreover, the community type–III was characterized by a dense crown cover dominated by *P. wallichiana* as the dominant tree species. However, in community type–I, *A. altissima* was the second highest crown forming the crown cover with a total area 279 m^2^/ha^–^^1^ (Table 6).

**Table 6:** Tree density/ha–^1^ and crown density/ha–^1^ of the plant community types based on Ward’s agglomerative clustering results.

Species	Groups					
	I ^D/Ha^	I ^C/Ha^	II ^D/Ha^	II ^C/Ha^	III ^D/Ha^	III ^C/Ha^
*AI*	0 ± 0	0 ± 0	0 ± 0	0 ± 0	1.42 ± 3.77	2.8 ± 7.41
*AA*	38.75 ± 30.9	51.46 ± 46.4	50 ± 62.53	42.42 ± 43.87	0 ± 0	0 ± 0
*CA*	0 ± 0	0 ± 0	0 ± 0	0 ± 0	2.85 ± 7.55	2.15 ± 5.69
*DS*	58.75 ± 124.9	31.76 ± 75.3	9 ± 28.46	3.73 ± 11.81	0 ± 0	0 ± 0
*DN*	1.25 ± 3.53	1.89 ± 5.37	0 ± 0	0 ± 0	0 ± 0	0 ± 0
*FG*	28.75 ± 56.42	7.04 ± 13.38	0 ± 0	0 ± 0	0 ± 0	0 ± 0
*FP*	16.25 ± 22.63	16.02 ± 23.4	31 ± 45.57	30.92 ± 48.77	10 ± 22.36	9.07 ± 20.28
*JR*	2.5 ± 7.07	31.15 ± 88.1	0 ± 0	0 ± 0	0 ± 0	0 ± 0
*MA*	3.75 ± 7.44	7.19 ± 14.12	0 ± 0	0 ± 0	0 ± 0	0 ± 0
*PR*	11.25 ± 22.32	32.59 ± 67.5	127 ± 135.97	373.07 ± 448.2	0 ± 0	0 ± 0
*PW*	1.25 ± 3.53	0.09 ± 0.26	0 ± 0	0 ± 0	70 ± 49.66	372.35 ± 659.89
*PA*	1.25 ± 3.53	1.41 ± 4	0 ± 0	0 ± 0	0 ± 0	0 ± 0
*PP*	3.75 ± 7.44	3.4 ± 6.74	0 ± 0	0 ± 0	0 ± 0	0 ± 0
*QG*	37.5 ± 58.97	37.76 ± 67.7	0 ± 0	0 ± 0	1.42 ± 3.77	1.61 ± 4.28
*QI*	0 ± 0	0 ± 0	8 ± 25.29	8.59 ± 27.17	97.14 ± 67.7	108.52 ± 71.44
*RP*	38.75 ± 82.19	16.19 ± 32.3	54 ± 77.34	17.48 ± 28.12	0 ± 0	0 ± 0
*ZA*	1.25 ± 3.53	1.57 ± 4.44	0 ± 0	0 ± 0	0 ± 0	0 ± 0
**Total**	**245**	**279**	**183**	**239.52**	**26**	**496.5**

## 4 Discussions

Despite having low covered areas than the desired 25.0 %, Pakistan also has a unique floral diversity with 6000 known plant species. Occurrence of diverse flora is primarily due to soil type i.e., Silt loam, loam, sandy loam, silty clay loam and clay loam (Ul Haq et al., 2022), climatic conditions (subtropical in north, moderate at foothills of mountain, tropical in desert in center-south), and topography (e.g., forests, highlands, river plains, desert areas, plateau, salt ranges, valleys, cultivated fields, and sistan basins) that range from sea level to 8611 masl (Ali and Khan, 2022, Ali et al., 2022, Khan et al., 2023). A significant number of floral species constituted the Pakistan's different forests, including mangrove forest (Khan et al., 2020) alpine forest, subalpine forest, Himalayan moist-temperate conifer forest, Himalayan dry-temperate conifer forest, subtropical chir pine forest, scrub forest, tropical thorn forest, riverine forest, and irrigate plantation (Khan et al., 2015). The present study provides a comprehensive assessment of floristic structure and composition, edaphic factors and floral community types of the Himalayan ‘Ecotone, temperate conifer forest in Swat district, Pakistan. A diverse flora (93 species) encompassing of trees, shrubs, grasses; herbs and ferns demonstrating (31 families) were identified in the study area. The conifer tree species were highly valuable species having high economic value, grown at higher elevation. Asteraceae, Poaceae, and Fabaceae were most prevalent under–storey vegetation, while Moraceae, Rosaceae, and Fagaceae were dominant tree families. According to the current findings, the floral structure and composition, vegetation community types were consistent with recent conducted studies, such as; Ali et al. (2019) and Rahman et al. (2021).

Ward's agglomerative clustering technique is widely accepted by ecologists’ method to classify the important value index of forest vegetation (Ullah et al., 2023, Rahman et al., 2021). By using importance value index, 17 woody tree species were divided into three various communities, i.e. type–I, type–II and type–III. *A. altissima* and *Q. glauca* dominated community type–I, while *P. roxburghii* and *A. altissima* dominated community type–II. *Q. incana*, and *P. wallichiana* co–dominant community type–III. *D salicifolia, R. pseudoacacia, F. palmata, Aesculus indica, C. australis, M. azedarach*, and others were associated with dominant species. According to several previous studies (Ali et al., 2019, Rahman et al., 2023) the current species holds a dominant position in the studied area. The area has recently been planted with trees such as *P. roxburghii, A. altissima*, and *M azedarach*, which will provide a beneficial environment for these communities in the future. In addition to fruiting trees, exotic species should be discouraged for a healthy ecosystem (Bennett et al., 2021).

The outcome of this study illustrated that the floral layers of community types–III has occupied higher altitude ranges (average = 1823.8 masl), while the floral layer of community types–II occupied the lower elevational ranges (average = 1263.8 m). There is evidence that topographic variables like elevation, slope, and aspect play a significant role in constituting the floral structure, composition (Muhammad et al., 2023), and function of Himalayan ‘Ecotone’ temperate conifer forest (Zhang et al., 2021). However, the floral layer of the community type–II of Himalayan ‘Ecotone’ temperate conifer forest has occupied the lower altitudes exhibited more compact flora and high temperatures. As a result of easy accessibility and intense anthropogenic interventions, such as grazing, this phenomenon may be observed in the study area (Nawaz et al., 2013). Due to the direct relationship between soil moisture contents, pH, and electrical conductivity, soil moisture, pH, and electrical conductivity also influenced vegetation distribution Himalayan ‘Ecotone’ temperate conifer forest (Nakhoul et al., 2020). Our results also revealed that the differences in moisture contents and soil pH were the resultant communities. The composition of vegetation and structure in Himalayan ‘Ecotone’ temperate conifer forest were influenced by soil physiochemical properties, such as organic matter and nutrient contents.

For quantifying the relationship between vegetation abundance, environmental variables, and edaphic factors, principal component analysis (PCA), redundancy analysis (RDA), and Canonical correspondence analysis (CCA) has been widely used (Ali et al., 2022, Ali and Khan, 2022, Sîrbu et al., 2022). In order to determine the most influential variables, we performed CCA-ordination, which explained 68.5 % of the variance in the data. The findings demonstrated that environmental factors such as elevation, soil pH, temperature, and canopy cover were significant factors which constituted vegetation composition. Likewise, it also has been reported that these are key factors that significantly influence vegetation growth and distribution (Ali et al., 2022, Ali and Khan, 2022, Sîrbu et al., 2022, Chen et al., 2023). According to the CCA–ordination biplot, vegetation and topographic variables on axis 1 showed a significant correlation. Elevation has been reported to affect the distribution patterns of forest vegetation (Ali et al., 2019).

The outcome of the study also highlighted that edaphic variables significantly effects on vegetation structure and composition. The mean soil pH in the study area was slightly acidic. Due to acidic soil pH the study area has harbored the higher abundance of conifer tree species. It has been known that acidic soils promote the growth of the conifers tree species (Neina, 2019). In addition, soil temperature also plays a significant role in constituting the composition of vegetation and distribution. The mean soil temperature in the study area was 11.2 °C, which is consistent with the humid and cool climate. Cool and humid conditions are favorable for the growth of broadleaf forests in the study area. Hence, the study area also bestowed a variety of broadleaf tree species.

## 5 Conclusions

The present study has provided a valuable insight into the floristic composition, structure, and correlation ship among vegetation, environmental, and edaphic variables in Himalayan ‘Ecotone, temperate conifer forest. Using the study's results and it recommended this ecosystem can be protected. In order to conserve natural habitats and maintain ecological integrity, conservation efforts should be directed towards preserving them and declared as biodiversity hotspot because of its high species richness and diversity.

## 6 Contribution of authors

F. Ali was involved in the data curation, writing original draft and formal analysis. M. Zeb did investigation, and methodology. M. Amin was involved in the conceptualization, and supervision. M.N.Rajpar writing, review and edit the paper and also provide resources. S. Hidayat perform visualization and validation. W.R.Khan helps in funding acquisition, project administration and software.

## Funding

The authors would like to extend their sincere appreciation to the Researchers Supporting Project Number (GP–IPM/2–23/9750500) Geram Putra GP–IPM Universiti Putra Malaysia.

## CRediT authorship contribution statement

**F. Ali:** Data curation, Writing – original draft, formal analysis. **M. Zeb:** Investigation, Methodology. **M. Amin:** Conceptualization, Supervision. **M.N. Rajpar:** Writing – review & editing, Resources. **S. Hidayat:** Visualization, validation. **W.R. Khan:** Funding acquisition, Project administration, Software.
